# Efficacy and safety of Chinese herbal medicine on blood glucose fluctuations in patients with type 2 diabetes mellitus: A protocol of systematic review and meta-analysis

**DOI:** 10.1097/MD.0000000000021904

**Published:** 2020-08-21

**Authors:** Yulin Leng, Xiujuan Zhou, Ziyan Xie, Zhipeng Hu, Hong Gao, Xiaoke Liu, Hongyan Xie, Xiaoxu Fu, Chunguang Xie

**Affiliations:** Hospital of Chengdu University of Traditional Chinese Medicine, Chengdu, Sichuan Province, China.

**Keywords:** blood glucose fluctuations, Chinese herbal medicine, protocol, systematic review, type 2 diabetes mellitus

## Abstract

**Background:**

As one of the major chronic diseases that seriously threaten human health, type 2 diabetes mellitus (T2DM) has become a global public health problem. Blood glucose fluctuation is a risk factor independent of hyperglycemia. At present, the measures to treat blood glucose fluctuations in patients with T2DM are insufficient in effectiveness and safety. Medical practice and clinical studies have proved that Chinese herbal medicine has obvious advantages in reducing blood glucose fluctuations. In this systematic review, we will assess the efficacy and safety of Chinese herbal medicine in the treatment of blood glucose fluctuations in patients with T2DM.

**Methods:**

We will search related literature of PubMed, Embase, Cochrane Library, Web of Science, China Biology Medicine Database, China National Knowledge Infrastructure, China Science and Technology Journal Database, and Wanfang Database, and will manually search grey documents such as literature such as conference articles and references articles. Eligible randomized controlled trials will be screened based on inclusion criteria, and data extraction, risk of bias assessment, publication bias assessment, subgroup analysis, and quality assessment will be performed. Review Manager version 5.3 software and stata version 13 software will be used for data analysis. Each process is independently conducted by 2 researchers, and if there is any objection, it will be submitted to the third researcher for resolution.

**Results:**

This study will provide evidence for the efficacy and safety of Chinese herbal medicine in the treatment of blood glucose fluctuations in patients with T2DM. Outcome measures include mean amplitude of glycemic excursions, 24 hours mean blood glucose, standard deviations of blood glucose, mean of daily differences, coefficient of variation, glucose time in range, fasting blood glucose, 2 hours postprandial blood glucose, glycated hemoglobin, HOMA-β, HOMA-IR, quality of life questionnaire, traditional Chinese medicine syndrome score, and adverse event.

## 1 Introduction

The diabetes atlas released by the International Diabetes Federation shows that as of 2019, there are about 463 million people with Diabetes worldwide.^[1]^ Taking China as an example, according to the assessment of the International Diabetes Federation, China's diabetes-related health expenditure is 294.6 billion dollars,^[1]^ which is a significant increase compared with 110 billion dollars assessed in 2017.^[2]^ Diabetes imposes a huge medical and economic burden on individuals and societies. Type 2 diabetes mellitus (T2DM) patients are prone to fluctuating hyperglycemia due to factors such as the progressive decline in islet β-cell function, impaired blood glucose regulation mechanisms, and poor treatment compliance.^[3–5]^ A number of studies have found that glucose fluctuation is an independent risk factor for T2DM complications such as macrovascular disease.^[6,7]^ Fluctuating hyperglycemia can promote inflammation and induce oxidative stress, and its damage degree to vascular endothelial function and atherosclerosis is greater than stable hyperglycemia.^[8–10]^ Compared with diabetic patients whose coefficient of variation of fasting blood glucose is less than 16%, patients with coefficient of variation of fasting blood glucose of more than 29% have a 2.68 times higher relative risk of developing diabetic retinopathy.^[11]^

The fundamental measure to reduce blood glucose fluctuation is to improve islet function.^[12,13]^ At present, the methods of medicine for treating T2DM mainly include oral drugs such as metformin, α-glucosidase inhibitors, dipeptidyl peptidase-4 inhibitors, and injectable drugs such as insulin and glucagon-like peptide-1 receptor agonists. These drugs have a good effect on reducing blood glucose values, but the improvement of islet function is limited. Besides, the stricter the blood glucose control, the higher the incidence of hypoglycemia events and the greater the fluctuation range of blood glucose. Therefore, the regulation of blood glucose homeostasis is currently the focus and difficulty of intervening in T2DM and complications.^[14]^ It is necessary to find therapeutic measures to effectively regulate blood glucose homeostasis and improve islet function.

In China, many patients with T2DM take Chinese herbal medicine to reduce blood glucose fluctuations. The characteristic of Chinese herbal medicine to regulate blood glucose fluctuations is to correct the internal environment instability caused by pathogenic factors and restore the body to a steady state. Its mechanism involves many pathways and targets, such as inhibiting islet β-cell apoptosis, regulating islet microcirculation.^[15,16]^ For example, Shenqi compound, a Chinese herbal medicine, can steadily reduce blood glucose of patients, relieve inflammation, and provide patients with quality of life.^[17,18]^ Researchers have conducted many randomized controlled trials (RCTs) on treatment of blood glucose fluctuations in patients with T2DM by Chinese herbal medicine, and there is still a lack of systematic evaluation on this topic. Therefore, in order to provide evidence-based evidence for Chinese herbal medicine to relieve blood glucose fluctuations, it is necessary to make a standard judgment on the efficacy and safety of Chinese herbal medicine in treatment of blood glucose fluctuations.

## 2 Methods

### 2.1 Study registration

This protocol has been registered on Open Science Framework grant number 10.17605/OSF.IO/T76EH (https://osf.io/t76eh). This report will be based on the preferred reporting items for systematic review and meta-analysis protocols.^[19]^

### 2.2 Eligibility criteria

#### 2.2.1 Study design

All RCTs of efficacy and safety of Chinese herbal medicine in the treatment of blood glucose fluctuations will be included in this systematic review.

#### 2.2.2 Participants

Studies included adult patients with T2DM. The diagnostic criteria for T2DM are in accordance with those proposed by the World Health Organization, or American Diabetes Association, or Chinese Diabetes Association.^[20–22]^ There are no restrictions on gender, nationality, course of disease, and complications of T2DM.

#### 2.2.3 Interventions

Taking any dosage of Chinese herbal medicine combined with basic conventional treatment as the treatment group, such as Chinese herbal medicine compound and single herbs. Excluding acupuncture, massage therapy, and other Traditional Chinese Medicine (TCM) non-drug therapies. Participants who did not received placebo or only basic conventional treatment but no Chinese herbal medicine will be used as the control group.

#### 2.2.4 Outcomes

##### 2.2.4.1 Primary efficacy outcomes

Blood glucose fluctuations will be set as primary efficacy outcomes, including mean amplitude of glycemic excursions, 24 hours mean blood glucose, standard deviations of blood glucose, mean of daily differences, coefficient of variation, glucose time in range, and so on.

##### 2.2.4.2 Secondary efficacy outcomes

Secondary efficacy outcomes include fasting and 2 hours postprandial blood glucose, glycated hemoglobin, islet function index (such as HOMA-β and HOMA-IR), quality of life questionnaire (such as short form-36 scale and adjusted diabetes-specific quality of life scale), and TCM syndrome score.

##### 2.2.4.3 Safety outcomes

Adverse event, such as hypoglycemia.

### 2.3 Study search

Electronic databases include PubMed, Embase, Cochrane Library, Web of Science, China Biology Medicine Database, China National Knowledge Infrastructure, China Science and Technology Journal Database, and Wanfang Database. The search terms include T2DM, blood glucose fluctuations, TCM, Chinese herbal medicine, and RCT. The search date is from establishment of the database to June 30, 2020. No restrictions on language. Besides, Clinical trial registries such as the Chinese Clinical Trial Registry, the Netherlands National Trial Register, and ClinicalTrials.gov, will be searched for ongoing studies. We will look for additional studies that may be eligible from the conference articles and references of articles identified for inclusion.

### 2.4 Study selection

**Figure 1:**
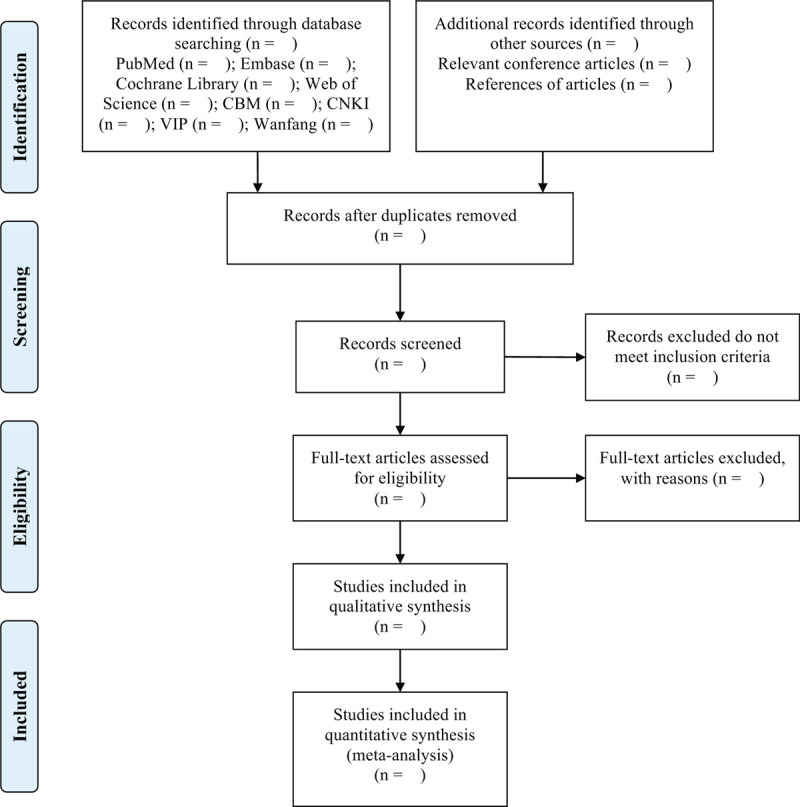
Flow chart of study selection process.

We will export the documents retrieved from the database to EndNote X9 software, and will use it to manage and delete duplicates. The retrieved studies will be screened by 2 researchers according to pre-established inclusion criteria. The full text will be screened to determine the eligible research. The above work process is carried out independently. If there is any objection, it will be resolved by the third researcher (Fig. 1).

### 2.5 Data extraction

Two researchers will independently extract data in pre-designed format, including article title, publication time, country or region, name of first author, funding, and demographic characteristics of the subjects, sample size, interventions in the treatment group and control groups, course of treatment, severity of disease, efficacy outcome and adverse events, data analysis strategy, and so on.

If the original data is insufficient or missing, the first author or corresponding author will be contacted by researchers via email and telephone. If missing data cannot be acquired, the study will only be included in narrative analysis. If there is any objection, it will be resolved by the third researcher.

### 2.6 Assessment of risk of bias

Risk of bias assessments, including random sequence generation, allocation concealment, blind methods, incomplete outcome data, selective outcome reports, and other biases, will be conducted by 2 researchers for all included studies. This work will be done using Cochrane collaboration tools.^[23]^ If there is any objection, it will be resolved by the third researcher.

### 2.7 Data analysis

The data analysis of this study will be conducted through Review Manager version 5.3 software and stata version 13 software. If the extracted data is continuous data, we will use the standard mean difference and the 95% confidence interval (CI) to evaluate. If it is dichotomous data, we will use risk ratio relative and 95% CI to represent. Chi-square test and *I*^2^ test will be used for heterogeneity assessment. If there is no heterogeneity (*P* > .05 and *I*^2^ ≤ 50%), the fixed effect model will be used for analysis.^[24]^

### 2.8 Assessment of heterogeneity

If studies have significant heterogeneity (*P* < .05 and *I*^2^ > 50%), we will use subgroup analysis and meta-regression.^[25]^ We established 3 hypotheses for the subgroup analysis: islet function, course of T2DM, and type of concomitant medication. Because blood glucose fluctuations are closely related to islet function, and there is a certain correlation between islet function and course of T2DM. Further evaluate credibility of the subgroup analysis based on the guidance for credible subgroup analysis.^[26,27]^

### 2.9 Quality assessment

In this study, we will refer to the lists used in previous meta-analysis to assess the quality of included studies. The quality of each study will be divided into high, medium, or low. Two researchers will independently assess quality based on the lists. If there is any objection, it will be resolved by the third researcher.

### 2.10 Assessment of publication bias

Funnel plots will be used to assess publication bias if more than 10 studies will be included in the meta-analysis. Begg and Egger tests will be used to assess the symmetry of the funnel plot with a boundary of *P*-value of .05.

### 2.11 Ethics

Because patient-specific data will not be used in this study, ethical approval is not required.

## 3 Discussion

At present, medical researchers and clinicians around the world have reached a consensus on the management of blood glucose in T2DM, that is, precisely reduce blood glucose and achieve control goals steadily. This goal reflects the importance of reducing blood glucose fluctuations. The advantage of western hypoglycemic drugs is that they can quickly and effectively reduce blood glucose, but the main factor of islet function decline has not been improved, leading to no definite effective relief of blood glucose fluctuations. TCM has a broad prospect in relieving blood glucose fluctuations and improving islet function. Therefore, we will implement this systematic review and meta-analysis to provide clinicians with high-quality evidence. This study only included RCTs, which may improve the quality of evidence but may lead to incomplete clinical studies being included.

## Author contributions

**Conceptualization:** Yulin Leng, Chunguang Xie, Xiaoxu Fu.

**Data curation:** Xiujuan Zhou, Ziyan Xie, Hong Gao.

**Formal analysis:** Yulin Leng, Xiaoke Liu, Hongyan Xie.

**Funding acquisition:** Chunguang Xie, Xiaoxu Fu.

**Methodology:** Yulin Leng, Xiujuan Zhou, Ziyan Xie.

**Project administration:** Chunguang Xie.

**Resources:** Chunguang Xie, Xiaoxu Fu.

**Software:** Yulin Leng, Zhipeng Hu.

**Supervision:** Chunguang Xie.

**Writing – original draft:** Yulin Leng.

**Writing – review & editing:** Yulin Leng, Chunguang Xie, Xiaoxu Fu.
